# UniBioDicts: Unified access to Biological Dictionaries

**DOI:** 10.1093/bioinformatics/btaa1065

**Published:** 2021-01-04

**Authors:** John Zobolas, Vasundra Touré, Martin Kuiper, Steven Vercruysse

**Affiliations:** Department of Biology, Norwegian University of Science and Technology (NTNU), NO-7491 Trondheim, Norway; Department of Biology, Norwegian University of Science and Technology (NTNU), NO-7491 Trondheim, Norway; Department of Biology, Norwegian University of Science and Technology (NTNU), NO-7491 Trondheim, Norway; Department of Biology, Norwegian University of Science and Technology (NTNU), NO-7491 Trondheim, Norway

## Abstract

**Summary:**

We present a set of software packages that provide uniform access to diverse biological vocabulary resources that are instrumental for current biocuration efforts and tools. The Unified Biological Dictionaries (UniBioDicts or UBDs) provide a single query-interface for accessing the online API services of leading biological data providers. Given a search string, UBDs return a list of matching term, identifier and metadata units from databases (e.g. UniProt), controlled vocabularies (e.g. PSI-MI) and ontologies (e.g. GO, via BioPortal). This functionality can be connected to input fields (user-interface components) that offer autocomplete lookup for these dictionaries. UBDs create a unified gateway for accessing life science concepts, helping curators find annotation terms across resources (based on descriptive metadata and unambiguous identifiers), and helping data users search and retrieve the right query terms.

**Availability and implementation:**

The UBDs are available through npm and the code is available in the GitHub organisation UniBioDicts (https://github.com/UniBioDicts) under the Affero GPL license.

**Supplementary information:**

Supplementary data are available at *Bioinformatics* online.

## 1 Motivation

The plethora of ontology terms and biological entity identifiers (IDs) provides a vast resource for use in annotations (by curators) and in database queries (by life scientists and computers), but specifying and finding them requires extensive navigation through an intimidating number of web resources and look-up forms. A universal way to perform a comprehensive search of life science databases, ontologies and vocabularies, supported by an autocomplete function that allows users to choose from a list of candidate terms with defining metadata, will greatly streamline this process. In addition, it will help to eliminate errors that stem from typing these terms manually without autocomplete support or options for semantic input checking. Furthermore, a unified lookup utility makes terms from diverse vocabularies easy to place together into context-rich annotations. The Visual Syntax Method (VSM) for example (Vercruysse and Kuiper, 2020), a technology that allows the flexible annotation of virtually any type of contextual information, can take advantage of unified access to such a large diversity of terms, e.g. in applications like causalBuilder (Touré et al., 2020). For these reasons, we set out to create a software suite that maps many of the diverse resources to a single data access and representation form.

## 2 Implementation

Each UBD module is an interface to an online server that provides ontology or controlled vocabulary data. A single dictionary module may provide access to one or several apparent ‘sub-dictionaries’; e.g. the BioPortal UBD presents each of its many combined biological-domain ontologies as a distinct sub-dictionary. When a UBD receives a request for data, it makes a custom request to the associated server’s API, and translates received data back into the format specified by the generic dictionary interface.

### 2.1 Main methods and data-types

Each UBD module offers the following methods to access a resource’s data, along with options for filtering, sorting and paging of results:

1. getDictInfos: returns a list of dictInfo objects which each hold information about one sub-dictionary of the data resource.
2. getEntries: returns entry objects. Each entry represents all relevant information about a specific biological concept. It is the combination of a computer-processable ID, at least one human-friendly term (a word or word sequence), and various metadata. The combined metadata makes it possible to inform curators of what a concept represents and how its meaning differs from others. For example, the UniProt UBD returns the ‘tp53’ concept via the standard properties: *id* (a URI, Uniform Resource ID: ‘https://www.uniprot.org/uniprot/P04637’), *terms* (a list: ‘P53_HUMAN’, ‘Cellular tumor antigen p53’, etc., with recommended name first and synonyms next), *descr* (text description of the protein), *dictID* (URI for the resource: ‘https://www.uniprot.org’); and an extra set of *z* sub-properties for data specific to UniProt: *z.species* (‘Homo Sapiens’), *z.genes* (‘TP53’, ‘P53’), etc.
3. getEntryMatchesForString: returns match objects. Each match combines one term-string (which may be a synonym, for one or several entries) with a specific entry that it represents. For example, querying the UniProt dictionary for ‘tumor antigen p53’ returns among others the above entry object for ‘tp53’, augmented with the property *str* (‘P53_HUMAN’).

For each UBD, these ‘get-’ methods have been harmonized with the associated resource’s available search and returned data. This is detailed in each UBD’s Readme on GitHub.

### 2.2 Additional features

1. Several UBDs are **optimized for curator use**: a match object’s *descr* and *str* are tweaked so that an autocomplete list can present available concepts in a way that is helpful in biocuration tasks. For example, when the Ensembl UBD queries its server for ‘tp53’, it receives several gene concepts with the same name and description, but different species and gene-synonyms. So to provide a more informative description, the last three are combined into an optimized *descr*.
2. Identifiers (*id*, *dictID*) are formed as **unambiguous, browsable URIs**. This supports giving users clickable access to details about a returned concept to verify if it conveys the desired semantics for their annotation (McMurry et al., 2017).
3. UBDs entry objects are **extensible**. Any extra information offered by a resource’s API can be added in the *entry.z* object, where it can later be used to customize or augment what an autocomplete shows to the user.

For further discussion on implementation and the expected impact of UBDs in the biocuration world, see Supplementary File S1.

## 3 Results

### 3.1 Implemented UBDs

Current UBDs map and unify the following biological resources and their respective APIs:

- BioPortal (Whetzel et al., 2011), the largest repository of biomedical ontologies, using the BioPortal REST API
- PubMed MEDLINE database of biomedical literature, using the Entrez programming utilities (Sayers, 2010)
- Noctua Entity Ontology, using their Solr Web service
- UniProt (The UniProt Consortium, 2019), using their REST API
- Ensembl (Zerbino et al., 2018)
- Ensembl Genomes (Howe et al., 2020)
- RNAcentral (The RNAcentral Consortium, 2018)
- Complex Portal (Meldal et al., 2019)

The last four UBDs each process a different data domain from the EBI Search API (Madeira et al., 2019). In addition, we provide a package that can combine several UBDs into one virtual dictionary, enabling the querying of multiple UBDs through one access point (see demo example where a vsm-box tool’s autocomplete is linked to UBDs).

### 3.2 Potential users

1. **Research software engineers** who use UBDs as a meta-API. They can programmatically access multiple resources in a uniform way and avoid dealing with disparate APIs that all have different documentation, specifications and data formats.
2. **Software developers** who build a project-specific curation tool. They can create input fields that offer autocomplete lookup in any set of UBDs and present matching terms and IDs in a selection panel. This is easily achieved by linking any dictionary to our reusable autocomplete web-component. UBDs can also be linked to a vsm-box (Vercruysse et al., 2020) to build curation applications, like causalBuilder.
3. **Biocurators** who use the above curation tools to find the terms they need. Autocomplete-based annotation allows biocurators to curate papers more quickly, conveniently and precisely, without having to copy text and IDs from elsewhere (Ward et al., 2012).

## Supplementary Material

btaa1065_Supplementary_Data
