# Supplementary material for: UniBioDicts: Unified access to Biological Dictionaries
Source: Bioinformatics. 2021 Jan 4;37(1):143–4. doi: 10.1093/bioinformatics/btaa1065 (PMC8034525; doi:10.1093/bioinformatics/btaa1065)
Supplement: btaa1065_Supplementary_Data [file btaa1065_supplementary_data.pdf]

<u>Author's comment</u>: In this supplementary file, we discuss what UBDs can offer to the biocuration and systems biology world, and the problems that we faced and had to overcome towards this goal.

Discussion

Although many of the leading resources provide individual support for finding appropriate identifiers, terms and definitions for biological entities and concepts, an overarching function that spans all resources is not yet available. Such a utility, providing real-time access to terminology from diverse biological subdomains through a unified interface, enables the development of tools that build upon the collective information residing in these disparate domains. A unified access to the wealth of descriptive information forms an essential enabling part of computational, semantic systems biology. Continuing in this spirit, we have recently started building another UBD that connects with PubDictionaries (Kim et al. 2019), and we invite future collaborators to join our UniBioDicts GitHub organisation and help build a growing collection of client APIs serving biological dictionaries. The currently developed packages cover a diverse range of web-services, API-technologies and associated data-types, providing concrete examples that facilitate the development of additional UBDs, or for that matter, any other domain dictionaries that may need to access online databases or ontologies for curation.

In the process of building the UBDs, we had to consult with at least one developer from each data or API resource, in order to clarify, refine, and simplify both their and our documentation and specification details, which subsequently led to a better design of the software. For example, individual APIs return error objects in different ways, which prompted us to harmonize our error handling specification across all UBDs. In order to deliver robust software that will benefit its users and optimize software development efforts in the future, face-to-face discussions coupled with extensive Q&A email correspondence proved to be essential (Prlić and Procter 2012). Finally, we wish to emphasize the importance that proper documentation has in a healthy software development practice (Karimzadeh and Hoffman 2018), and its vital role in achieving our aforementioned goal.

References

Karimzadeh, Mehran, and Michael M. Hoffman. 2018. "Top Considerations for Creating Bioinformatics Software Documentation." *Briefings in Bioinformatics* 19 (4): 693–99.

Kim, Jin-Dong, Yue Wang, Toyofumi Fujiwara, Shujiro Okuda, Tiffany J. Callahan, and K. Bretonnel Cohen. 2019. "Open Agile Text Mining for Bioinformatics: The PubAnnotation Ecosystem." *Bioinformatics* 35 (21): 4372–80.

Prlić, Andreas, and James B. Procter. 2012. "Ten Simple Rules for the Open Development of Scientific Software." *PLoS Computational Biology* 8 (12): e1002802.
